# C-terminal deletion of NOTCH1 intracellular domain (N1^ICD^) increases its stability but does not amplify and recapitulate N1^ICD^-dependent signalling

**DOI:** 10.1038/s41598-017-05119-0

**Published:** 2017-07-11

**Authors:** Jennifer Blain, Jessily Bédard, Maureen Thompson, François-Michel Boisvert, Marie-Josée Boucher

**Affiliations:** 1 Gastroenterology Division, Department of medicine, Faculté de Médecine et des Sciences de la Santé, Pavillon de Recherche Appliquée sur le Cancer, Université de Sherbrooke, Sherbrooke, J1E 4K8 Canada; 2 Department of anatomy and cell biology, Faculté de Médecine et des Sciences de la Santé, Pavillon de Recherche Appliquée sur le Cancer, Université de Sherbrooke, Sherbrooke, J1E 4K8 Canada

## Abstract

Since the generation of a mouse strain conditionally expressing the active intracellular domain of Notch1 (N1^ICD^), many laboratories have exploited this model (Rosa^N1-ICD^) to assess the impact of constitutive Notch1 signalling activation in normal and pathological processes. It should be underscored that Cre-recombination leads to the expression of a C-terminally truncated form of N1^ICD^ (N1^ICDdC^) in the Rosa^N1-ICD^ mutant mice. Given that no studies were undertaken to delineate whether deletion of this region leaves intact N1^ICD^ function, stable cell lines with single targeted integration of inducible N1^ICD^ and N1^ICDdC^ were generated. We found that C-terminal deletion of N1^ICD^ stabilized the protein but did not promote the activity of Notch responsive promoters. Furthermore, despite higher expression levels, N1^ICDdC^ failed to phenocopy N1^ICD^ in the promotion of anchorage-independent growth. Our results thus suggest that the C-terminal region of N1^ICD^ plays a role in shaping the Notch response. Therefore, it should be taken into consideration that N1^ICD^ is truncated when interpreting phenotypes of Rosa^N1-ICD^ mutant mice.

## Introduction

The Notch pathway is a highly conserved signalling pathway with a relatively simple architecture^1, 2^. The transmembrane Notch receptors (NOTCH 1–4) undergo a series of proteolytic cleavages upon ligand binding releasing the Notch intracellular domain (N^ICD^). The N^ICD^ then translocates into the nucleus where, in association with CSL [CBF1, Su(H) and LAG-1] and MAML1 (Mastermind-like 1), forms a core transcriptional activation complex impacting on gene expression. The release of N^ICD^ thus constitutes a limiting step for activation of this signalling pathway devoid of amplification process. Although the precise mechanisms remain to be clarified, N^ICD^ turnover, consequent to its proteasomal degradation, dismantles the N^ICD^/CSL/MAML1 ternary complex and put an end to Notch activity *viz*. Notch-dependent gene regulation^3, 4^. The PEST domain, located C-terminally of N^ICD^, was shown to play a critical role in N^ICD^ turnover^4^.

The Notch signalling pathway orchestrates many developmental processes as well as ensures tissue homeostasis in the adult. Notably, aberrant Notch signalling is frequently observed in different cancer types^5, 6^ underscoring the need to maintain Notch signalling under tight regulation to preserve tissue homeostasis. To better define the impact of Notch activation in physiological or pathological contexts, mutant mouse with targeted insertion of mouse Notch1 intracellular domain (N1^ICD^) into the *GT(ROSA)26Sor* locus was generated (Rosa^N1-ICD^)^7^. This mouse strain is now available through The Jackson Laboratory and is used in conjunction with Cre-recombinase expressing strain to generate cell type/tissue-specific expression of N1^ICD^. Up to now, over 125 publications reported diverse phenotypes taking advantage of this Rosa^N1-ICD^ mouse strain. It is of particular note that the sequence encoding the mouse N1^ICD^ in the Rosa^N1-ICD^ model lacks the last C-terminal 238 amino acids^7^. Although the original paper did not explicitly provide reason as to why the entire N1^ICD^ coding sequence was not used, this Rosa^N1-ICD^ strain is exploited to generate mouse models with cell type/tissue-specific constitutive activation of Notch1 signalling.

It is becoming clear that the relatively simple architecture of the Notch signalling pathway must hide complex regulatory mechanisms contributing to the coordinated nuclear outcomes of the N^ICD^/CSL/MAML1 ternary complex^1, 2, 8, 9^. Previous studies have suggested that the N1^ICD^/CSL/MAML1 transcriptional platform is assembled in a precise stepwise manner^10, 11^ and other interacting factors most likely joined this platform^12^ to ensure efficient transcription of target genes. Accumulating evidence also support roles for post-transcriptional modifications such as phosphorylation^4, 10, 13–18^, acetylation^19, 20^, methylation^21^ and ubiquitination^20, 22^ in the coordinated assembly and disassembly of the Notch1-dependent transcriptional platform^1, 2, 8, 9^. Notably, methylation of Notch1 within its C-terminal domain recently appeared critical in dosing the Notch response^21^.

Given that, upon its release from the transmembrane receptor, N1^ICD^ undergoes sequential post-translational modifications such as phosphorylation on amino acids that remain to be identified^18, 23^, and that the C-terminal domain of N1^ICD^ potentially harbours sites targeted by phosphorylation, methylation and/or ubiquitination^8, 9^, this study was undertaken to determine whether a N1^ICD^ deleted of its C-terminal domain (N1^ICDdC^) can substitute for N1^ICD^ in functional studies. Herein, we provide evidence that despite higher expression levels, the transcriptional output of N1^ICDdC^ is distinct from N1^ICD^. Moreover, N1^ICDdC^ fails to phenocopy N1^ICD^ in promoting anchorage-independent growth. Therefore, given these discrepancies in function between N1^ICD^ and N1^ICDdC^, we should be careful when interpreting the functional impact of Notch1 activation on the basis of results obtained with models using a deleted version of N1^ICD^ such as the Rosa^N1-ICD^ mouse strain.

## Results

### Generation of inducible U2OS Flp-In^TM^ T-REx^TM^ cell lines expressing N1^ICD^ or N1^ICDdC^

**Figure 1:**
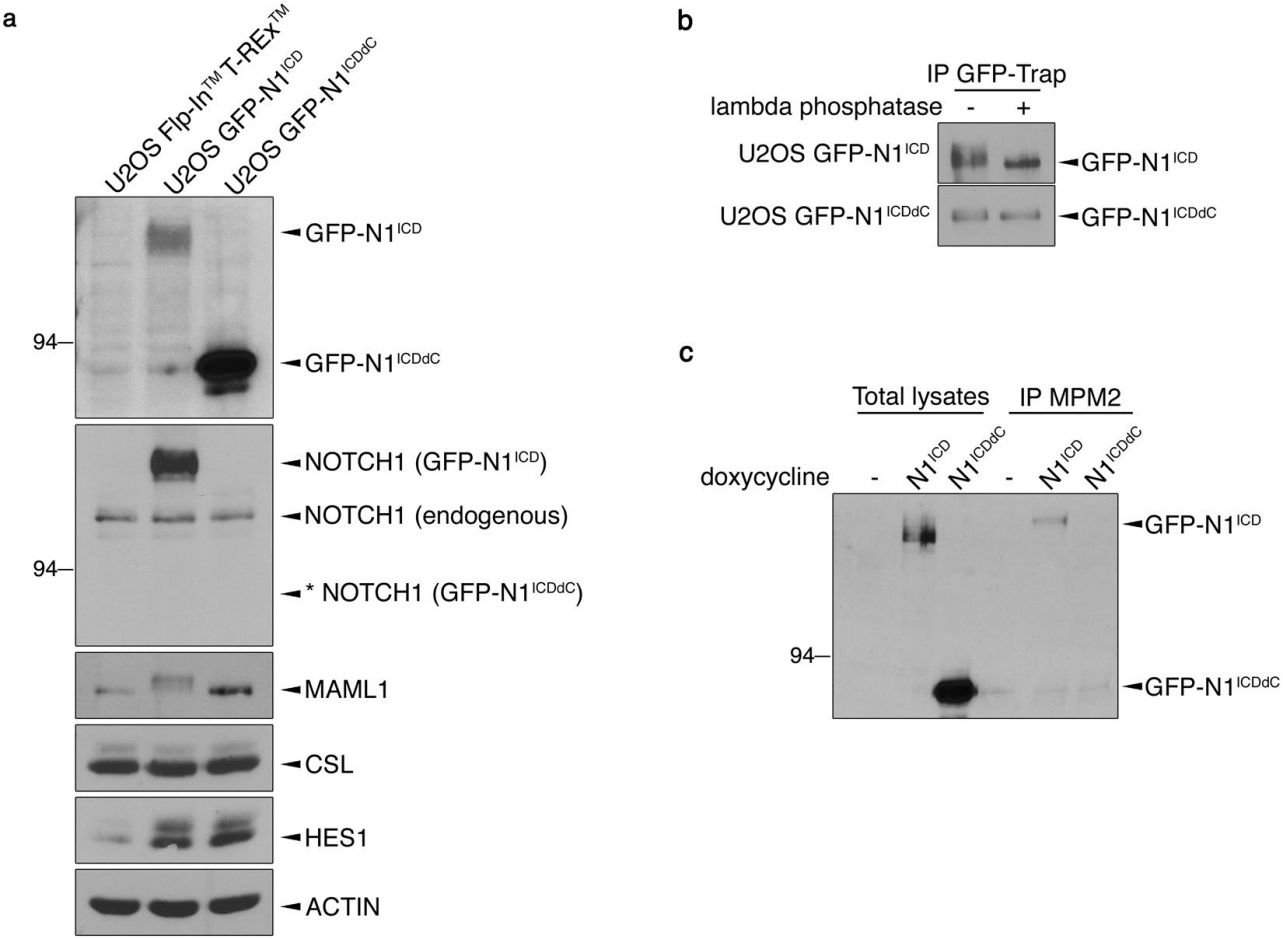
Generation of U2OS Flp-In^TM^ T-REx^TM^ cell lines expressing inducible GFP-N1^ICD^ or GFP-N1^ICDdC^. (**a**) The parental U2OS Flp-In^TM^ T-REx^TM^, stable U2OS N1^ICD^ and U2OS GFP-N1^ICDdC^ cell populations were treated for 24 h with doxycycline. Total cell lysates were analysed for GFP-N1^ICD^ and GFP-N1^ICDdC^ expression using an anti-GFP antibody. An anti-NOTCH1 antibody was used for detection of endogenous NOTCH1 and GFP-N1^ICD^. The asterisk * denotes the expected molecular weight of GFP-N1^ICDdC^ not detected by the anti-NOTCH1 antibody. Endogenous expression levels of MAML1, CSL, HES1 and ACTIN were analysed by immunoblotting using specific antibodies. Cropped blots are displayed and full-length blots are included in Supplementary Information. (**b**) U2OS GFP-N1^ICD^ and U2OS GFP-N1^ICDdC^ cells were treated for 24 h with doxycycline before being lysed and subjected to immunoprecipitation using GFP-Trap agarose beads (IP GFP-Trap). Phosphatase assays were performed ( + ) on half of the immunoprecipitate using lambda phosphatase. GFP-N1^ICD^ and GFP-N1^ICDdC^ expression were analysed using an anti-GFP antibody. Cropped blots are displayed and full-length blots are included in Supplementary Information. (**c**) Total cell lysates from uninduced U2OS GFP-N1^ICD^ (−) or 24 h doxycycline-induced U2OS GFP-N1^ICD^ (N1^ICD^) and U2OS GFP-N1^ICDdC^ (N1^ICDdC^) were prepared and phosphorylated proteins were immunoprecipitated using an anti-MPM2 antibody (IP MPM2). GFP-N1^ICD^ and GFP-N1^ICDdC^ expression were analysed using an anti-GFP antibody. Cropped blot are displayed and full-length blot is included in Supplementary Information.

To characterize the functional impact of deleting the C-terminal domain of N1^ICD^, stable cell lines expressing doxycycline inducible GFP-N1^ICD^ or GFP-N1^ICDdC^ were generated. We took advantage of the U2OS Flp-In^TM^ T-REx^TM^ cells in order to target GFP-N1^ICD^ and GFP-N1^ICDdC^ integration at a single transcriptionally active genomic locus^24^ and ensuring expression levels comparable to endogenous expression levels. The single targeted integration allowed minimizing for difference between GFP-N1^ICD^ and GFP-N1^ICDdC^ cell populations owing to variable integration sites. Of note, the encoded N1^ICDdC^ is the corresponding human sequence of the mouse N1^ICD^ inserted into the Rosa locus of the Rosa^N1-ICD^ mouse strain^7^. To confirm N1^ICD^ and N1^ICDdC^ expression in our stable U2OS Flp-In^TM^ T-REx^TM^ GFP- N1^ICD^ and U2OS Flp-In^TM^ T-REx^TM^ GFP- N1^ICDdC^ cell populations (thereafter named U2OS GFP-N1^ICD^ and U2OS GFP-N1^ICDdC^ respectively), cells were induced with doxycycline. Solely the U2OS GFP-N1^ICD^ and U2OS GFP-N1^ICDdC^ cells, and not the parental U2OS Flp-In^TM^ T-REx^TM^ cells, expressed a GFP fusion protein at the expected molecular weight (~140 kDa for GFP-N1^ICD^ and ~90 kDa for GFP-N1^ICDdC^) upon doxycycline exposure (Fig. 1a). In addition to recognizing the endogenous transmembrane NOTCH1 subunit, a NOTCH1 specific antibody detected the GFP-N1^ICD^ protein but not GFP-N1^ICDdC^ most likely owing to the loss of the C-terminal epitope on the GFP-N1^ICDdC^ protein. Of note, the endogenous NOTCH1 expression levels were not modulated by the concomitant expression of GFP-N1^ICD^ or GFP-N1^ICDdC^. Moreover, the N1^ICD^ interacting partners CSL and MAML1 were expressed at comparable levels in the U2OS cell populations although retarded migration on SDS-PAGE of MAML1 was regularly detected in induced U2OS GFP-N1^ICD^ cells (Fig. 1a). Increased expression levels of the Notch target HES1 were detected in the induced U2OS GFP-N1^ICD^ and GFP-N1^ICDdC^ cell populations suggesting that N1^ICD^ and N1^ICDdC^ are able to promote Notch signalling.

We previously provided evidence that endogenous N1^ICD^ is phosphorylated upon ligand-dependent and -independent NOTCH1 activation^18^. To test whether the expressed N1^ICD^ undergoes similar regulatory mechanisms in U2OS, phosphorylation levels of N1^ICD^ and N1^ICDdC^ were evaluated. Phosphorylation assays demonstrated that N1^ICD^ was efficiently phosphorylated in U2OS whereas N1^ICDdC^ was barely subjected to such a post-translational modification (Fig. 1b). Moreover, upon immunoprecipitation of proteins phosphorylated on serine or threonine residues using an anti-MPM2 antibody, only N1^ICD^ was detected by immunoblotting (Fig. 1c). These results suggest that, similarly to endogenous N1^ICD^ ^18, 23^, N1^ICD^ is post-translationally modified by phosphorylation whereas N1^ICDdC^ lacks regulatory phospho-sites.

**Figure 2:**
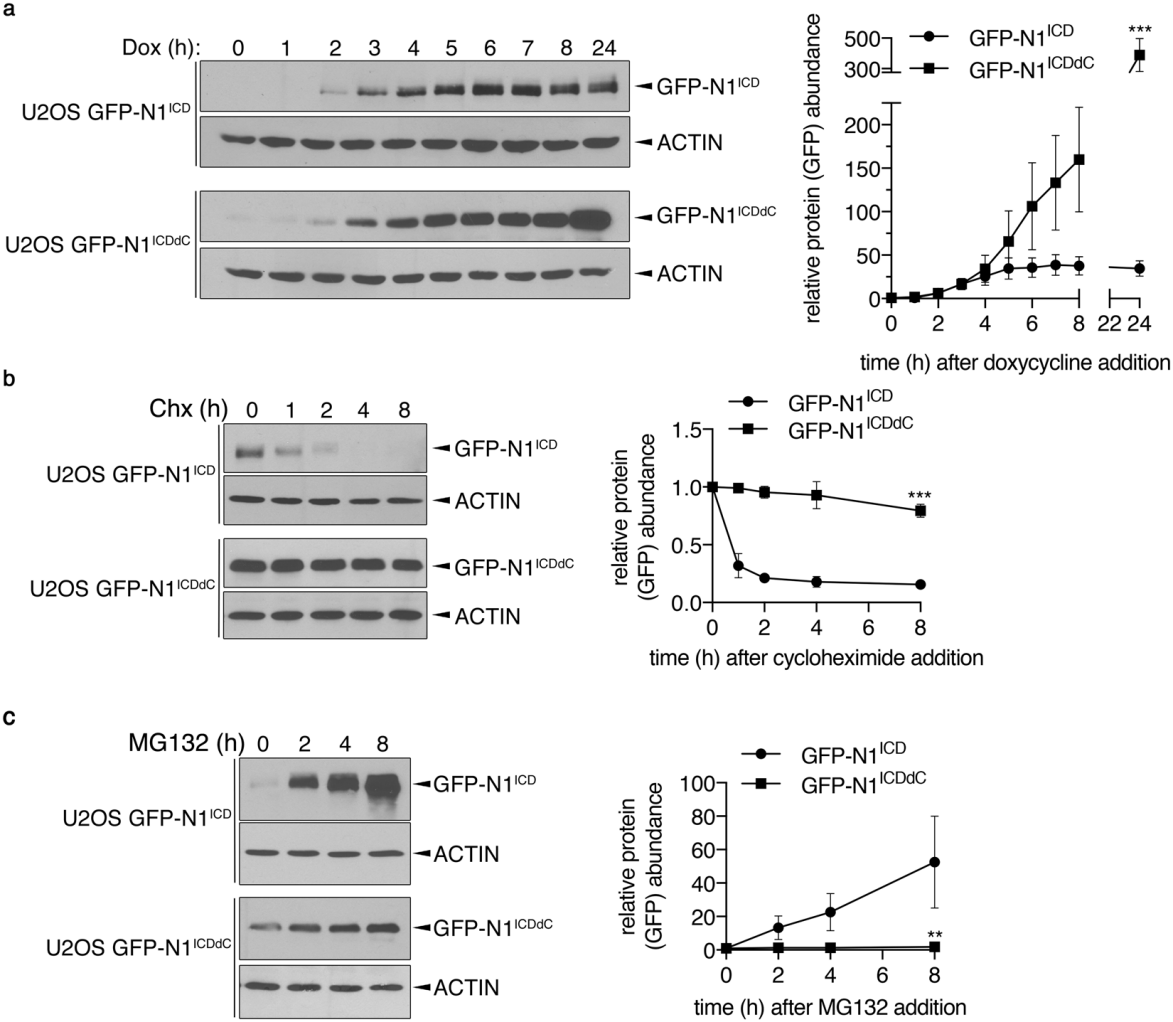
N1^ICDdC^ is more stable than N1^ICD^. (**a**) U2OS GFP-N1^ICD^ and U2OS GFP-N1^ICDdC^ cell populations were induced with doxycycline (Dox) for the indicated time period. GFP-N1^ICD^ and GFP-N1^ICDdC^ expression levels were analysed using an anti-GFP antibody. Representative immunoblots are shown. Cropped blots are displayed and full-length blots are included in Supplementary Information. A graphical representation of the mean GFP expression levels ± SEM of 5 independent experiments is shown where GFP expression levels were normalized to ACTIN and GFP/ACTIN ratio in uninduced cells was set at 1. ***p < 0.001 as compared with GFP-N1^ICD^. (**b**) U2OS GFP-N1^ICD^ and U2OS GFP-N1^ICDdC^ cell populations were induced with doxycycline for 24 h before being treated with cycloheximide (Chx) for the indicated time period. Representative immunoblots are shown. Cropped blots are displayed and full-length blots are included in Supplementary Information. A graphical representation of the mean GFP expression levels ± SEM of 4 independent experiments is shown where GFP expression levels were normalized to ACTIN and GFP/ACTIN ratio in untreated cells was set at 1. ***p < 0.001 as compared with GFP-N1^ICD^. (**c**) U2OS GFP-N1^ICD^ and U2OS GFP-N1^ICDdC^ cell populations were induced with doxycycline for 24 h before being treated with MG132 for the indicated time period. Representative immunoblots are shown. Cropped blots are displayed and full-length blots are included in Supplementary Information. A graphical representation of the mean GFP expression levels ± SEM of 5 independent experiments is shown where GFP expression levels were normalized to ACTIN and GFP/ACTIN ratio in untreated cells was set at 1. **p < 0.01 as compared with GFP-N1^ICD^.

Albeit using U2OS Flp-In^TM^ T-REx^TM^ cells for single targeted integration, greater expression levels of N1^ICDdC^ as compared to N1^ICD^ were consistently observed after prolonged doxycycline exposure (see Fig. 1a) suggesting post-transcriptionally events regulating protein expression. To dissect the N1^ICD^ and N1^ICDdC^ expression profile upon doxycycline addition, a time-dependent response was performed. As shown in Fig. 2a, N1^ICD^ expression reached a steady-state level upon 5–6 hours addition of doxycycline whereas N1^ICDdC^ expression continuously increased over time. Given that the C-terminal domain of N1^ICD^ was suggested to participate in protein turnover^4^, cycloheximide and MG132 treatment were carried out to evaluate N1^ICD^ and N1^ICDdC^ stability and proteasomal degradation. The expression of N1^ICD^ decreased upon cycloheximide addition (Fig. 2b) and increased upon MG132 treatment (Fig. 2c) suggesting that N1^ICD^ is relatively unstable most likely owing to its proteasome-dependent degradation. In opposition, N1^ICDdC^ was only slightly modulated by protein synthesis or proteasome inhibition (Fig. 2b and c). These results are thus in accordance with the reported requirement of the C-terminal domain for N1^ICD^ proteasomal turnover^4^. Altogether, we generated inducible U2OS cell populations expressing either GFP-N1^ICD^ or GFP- N1^ICDdC^. The expressed N1^ICD^ and N1^ICDdC^ display characteristics similar to what was previously reported i.e. increased stability of N1^ICDdC^ as compared to the rapid turnover of N1^ICD^.

### N1^ICDdC^ does not recapitulate N1^ICD^

**Figure 3:**
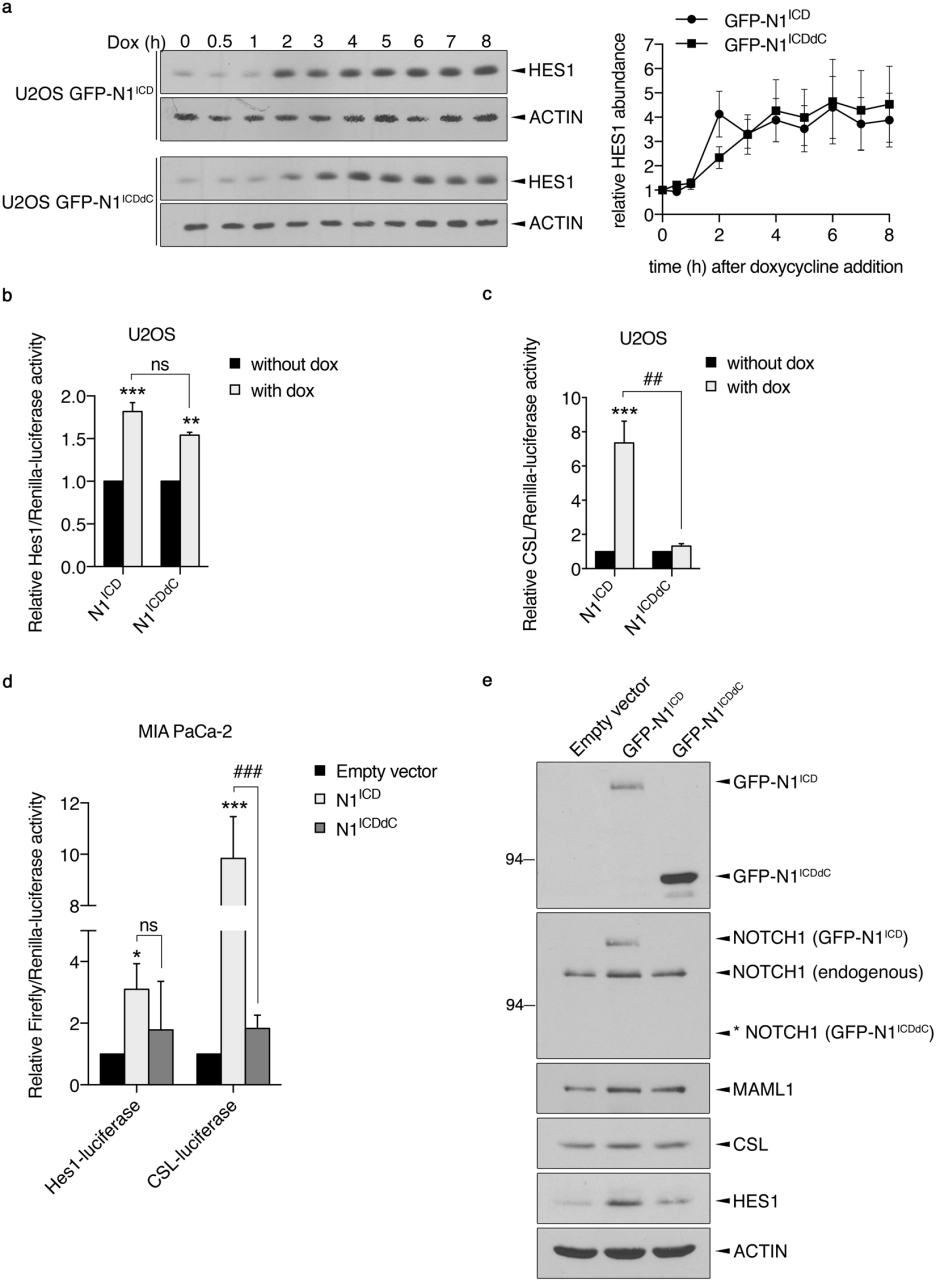
Elevated N1^ICDdC^ expression levels as compared to N1^ICD^ do not lead to higher expression of Notch responsive genes. (**a**) U2OS GFP-N1^ICD^ and GFP-N1^ICDdC^ cell populations were induced with doxycycline (Dox) for the indicated time period. HES1 and ACTIN expression levels were analysed using specific antibodies. Representative immunoblots are shown. Cropped blots are displayed and full-length blots are included in Supplementary Information. A graphical representation of the mean HES1 expression levels ± SEM of 4 independent experiments is shown where HES1 expression levels were normalized to ACTIN and HES1/ACTIN ratio in uninduced cells was set at 1. (**b**) Uninduced U2OS GFP-N1^ICD^ and GFP-N1^ICDdC^ cells were transfected with the Hes1-luciferase and Renilla-luciferase reporter constructs, and cells were left uninduced (without dox) or induced with doxycycline (with dox) for 24 h. The experiment was performed twice in triplicate. The data are expressed as the means ± SEM of Hes1-luciferase activity/Renilla-luciferase activity where the relative activity in uninduced cells (without dox) was set at 1. ***p < 0.001, **p < 0.01 as compared with uninduced cells. ns = not significant. (**c**) Uninduced U2OS GFP-N1^ICD^ and GFP-N1^ICDdC^ cells were transfected with the CSL-luciferase and Renilla-luciferase reporter constructs, and cells were left uninduced (without dox) or induced with doxycycline (with dox) for 24 h. The experiment was performed 4 times in triplicate. The data are expressed as the mean ± SEM of CSL-luciferase activity/Renilla-luciferase activity where the relative activity in uninduced cells (without dox) was set at 1. ***p < 0.001 as compared with uninduced cells. ##p < 0.01. (**d**) MIA PaCa-2 cells were transfected with pDEST53 (empty vector), pDEST53-N1^ICD^ (N1^ICD^) or pDEST53-N1^ICDdC^ (N1^ICDdC^) together with the Hes1-luciferase or CSL-luciferase and Renilla-luciferase reporter constructs. Luciferase activities were measured the following day. The experiment was performed 3 times in quadruplicate. The data are expressed as the mean ± SEM of Hes1-luciferase or CSL-luciferase activity/Renilla-luciferase activity where the relative activity in empty vector transfected cells was set at 1. *p < 0.05, ***p < 0.001 as compared with empty vector transfected cells. ###p < 0.001. ns = not significant. (**e**) MIA PaCa-2 cells were transfected with pDEST53 (empty vector), pDEST53-N1^ICD^ (GFP-N1^ICD^) or pDEST53-N1^ICDdC^ (GFP-N1^ICDdC^). The following day, total cell lysates were analysed for N1^ICD^ and N1^ICDdC^ expression using an anti-GFP antibody. An anti-NOTCH1 antibody was also used for detection of endogenous NOTCH1 and GFP-N1^ICD^. The asterisk * denotes the expected molecular weight of GFP-N1^ICDdC^ not detected by the anti-NOTCH1 antibody. Expression levels of MAML1, CSL, HES1 and ACTIN were analysed by immunoblotting using specific antibodies. Cropped blots are displayed and full-length blots are included in Supplementary Information.

Despite a much greater expression levels of N1^ICDdC^ as compared to N1^ICD^ upon prolonged doxycycline exposure, comparable HES1 expression levels were detected (Fig. 1a). In attempt to better discern the capacity of N1^ICD^ and N1^ICDdC^ to modulate Notch target(s), a kinetic of HES1 expression at shorter time point was performed given that N1^ICD^ and N1^ICDdC^ were expressed at comparable levels within the first 5 hours of doxycycline addition (Fig. 2a). No significant differences in HES1 expression levels were noted in U2OS GFP-N1^ICDdC^ as compared to U2OS GFP-N1^ICD^ cells upon doxycycline addition although U2OS GFP-N1^ICD^ cells appeared to achieve HES1 steady-state levels faster (2 h) than U2OS GFP-N1^ICDdC^ cells (4 h) (Fig. 3a). Luciferase assays also failed to unveil any significant difference in the ability of N1^ICD^ and N1^ICDdC^ to promote Hes1 promoter activity in U2OS (Fig. 3b). However, only N1^ICD^ significantly stimulated the activity of the Notch pathway responsive reporter CSL-luciferase (Fig. 3c). The CSL-luciferase reporter gene contains multimerized CSL DNA-binding site upstream of the luciferase gene reflecting more global Notch-dependent transcription as opposed to Hes1-luciferase that monitor activity only on the Hes1 promoter. Our results thus suggest that N1^ICD^ and N1^ICDdC^ may not be equipotent in modulating gene expression since the higher expression levels of N1^ICDdC^ was not converted into a higher transcriptional output or elevated expression of the Notch target HES1. To verify this in another system, transient transfection of N1^ICD^ and N1^ICDdC^ were performed in the pancreatic cancer cell line MIA PaCa-2. As opposed to N1^ICD^, N1^ICDdC^ was unable to significantly up-regulate the activity of the reporter genes Hes1-luciferase and CSL-luciferase in that cell model (Fig. 3d). Additionally, although expressed at much higher levels, N1^ICDdC^ was not as competent than N1^ICD^ to promote HES1 protein expression (Fig. 3e). Altogether, these results suggest that N1^ICD^ and N1^ICDdC^ may have distinct transcriptional potential.

**Figure 4:**
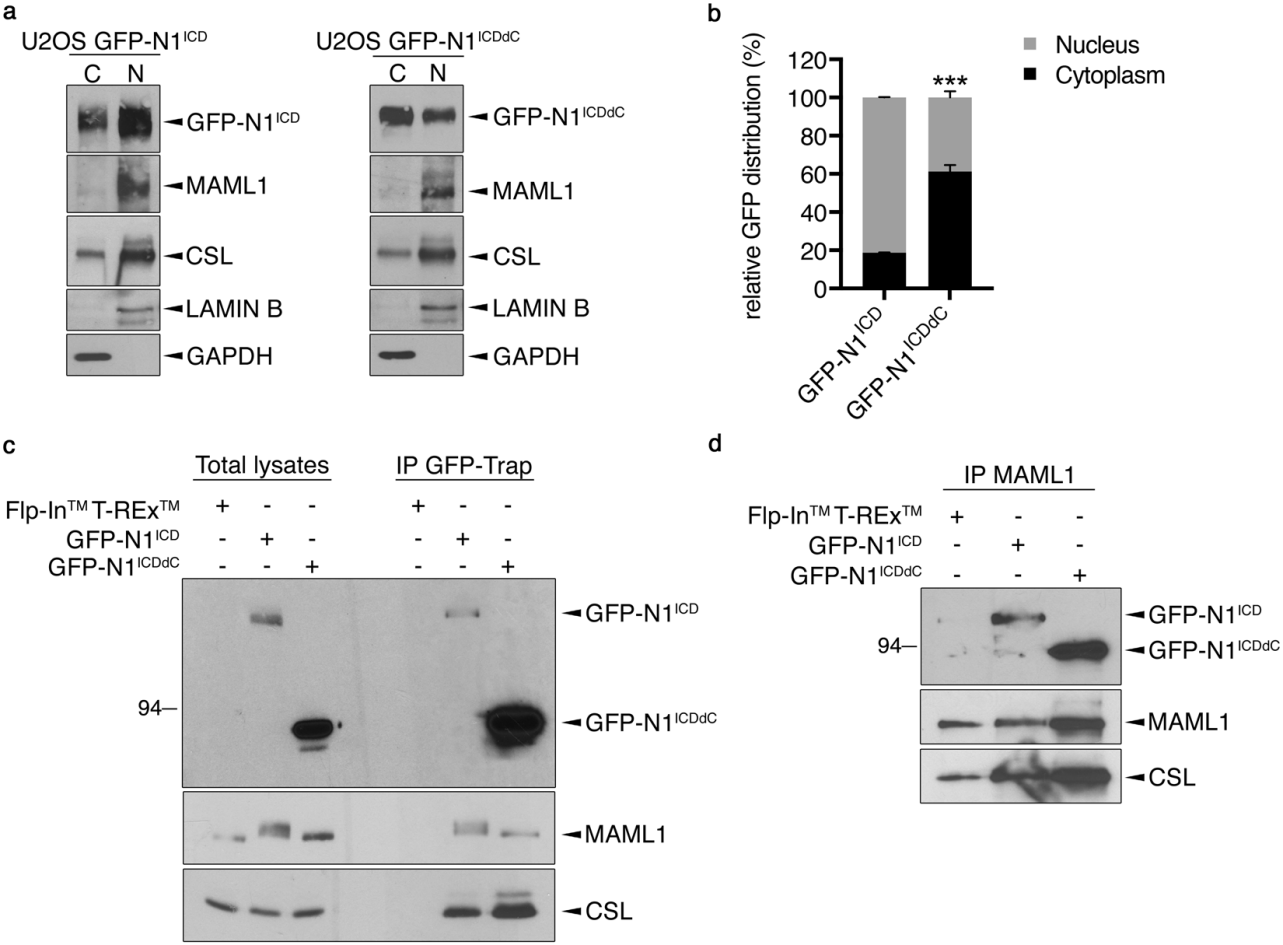
N1^ICDdC^ maintains its ability to interact with CSL and MAML1. (**a**) U2OS GFP-N1^ICD^ and GFP-N1^ICDdC^ cell populations were induced with doxycycline for 24 h. Cytosolic and nuclear proteins were fractionated and immunoblotting analyses were performed using the specified antibodies. GFP-N1^ICD^ and GFP-N1^ICDdC^ expression levels were analysed using an anti-GFP antibody. Cropped blots are displayed and full-length blots are included in Supplementary Information. (**b**) A graphical representation of the mean distribution of GFP between cytosolic and nuclear compartment ± SEM of 3 independent experiments. Representative immunoblots were shown in a. ***p < 0.001 as compared with GFP distribution in U2OS GFP-N1^ICD^ cells. (**c**) The parental U2OS Flp-In^TM^ T-REx^TM^, stable U2OS GFP-N1^ICD^ and GFP-N1^ICDdC^ cell populations were treated for 24 h with doxycycline. Total cell lysates were subjected to immunoprecipitation using GFP-Trap agarose beads (IP GFP-Trap) and analysed for N1^ICD^ and N1^ICDdC^ expression (using GFP antibody) as well as MAML1 and CSL. Cropped blots are displayed and full-length blots are included in Supplementary Information. (**d**) The parental U2OS Flp-In^TM^ T-REx^TM^, stable U2OS GFP-N1^ICD^ and GFP-N1^ICDdC^ cell populations were treated for 24 h with doxycycline. Total cell lysates were subjected to immunoprecipitation using an anti-MAML1 antibody (IP MAML1) and analysed for N1^ICD^ and N1^ICDdC^ expression (using GFP antibody) as well as MAML1 and CSL. Cropped blots are displayed and full-length blots are included in Supplementary Information.

Nuclear translocation of N1^ICD^ and its association with its transcriptional partners CSL and MAML1 are mandatory to impact on gene expression. Both N1^ICD^ and N1^ICDdC^ were detected within the nuclear compartment (Fig. 4a) although a smaller proportion of N1^ICDdC^ was found within the nucleus as compared to the 80% of N1^ICD^ localized within the nucleus (Fig. 4b). Of note, most likely due to its higher expression levels, expression levels of N1^ICDdC^ detected within the nucleus were at least as much as the N1^ICD^ nuclear expression levels (not shown). So, the amount of nuclear N1^ICDdC^ could not account for the reduced transcriptional capacity observed in luciferase assays (Fig. 3b and c). The altered subcellular distribution only affected N1^ICDdC^ as CSL and MAML1 were distributed in a similar manner in the U2OS GFP- N1^ICD^ and GFP-N1^ICDdC^ cell populations i.e. mainly in the nuclear compartment. Co-immunoprecipitation studies indicated that N1^ICDdC^ was still able to interact with CSL and MAML1 (Fig. 4c and d). Therefore, N1^ICDdC^ retains its capacity to localize within the nucleus and interact with its partners CSL and MAML1.

**Figure 5:**
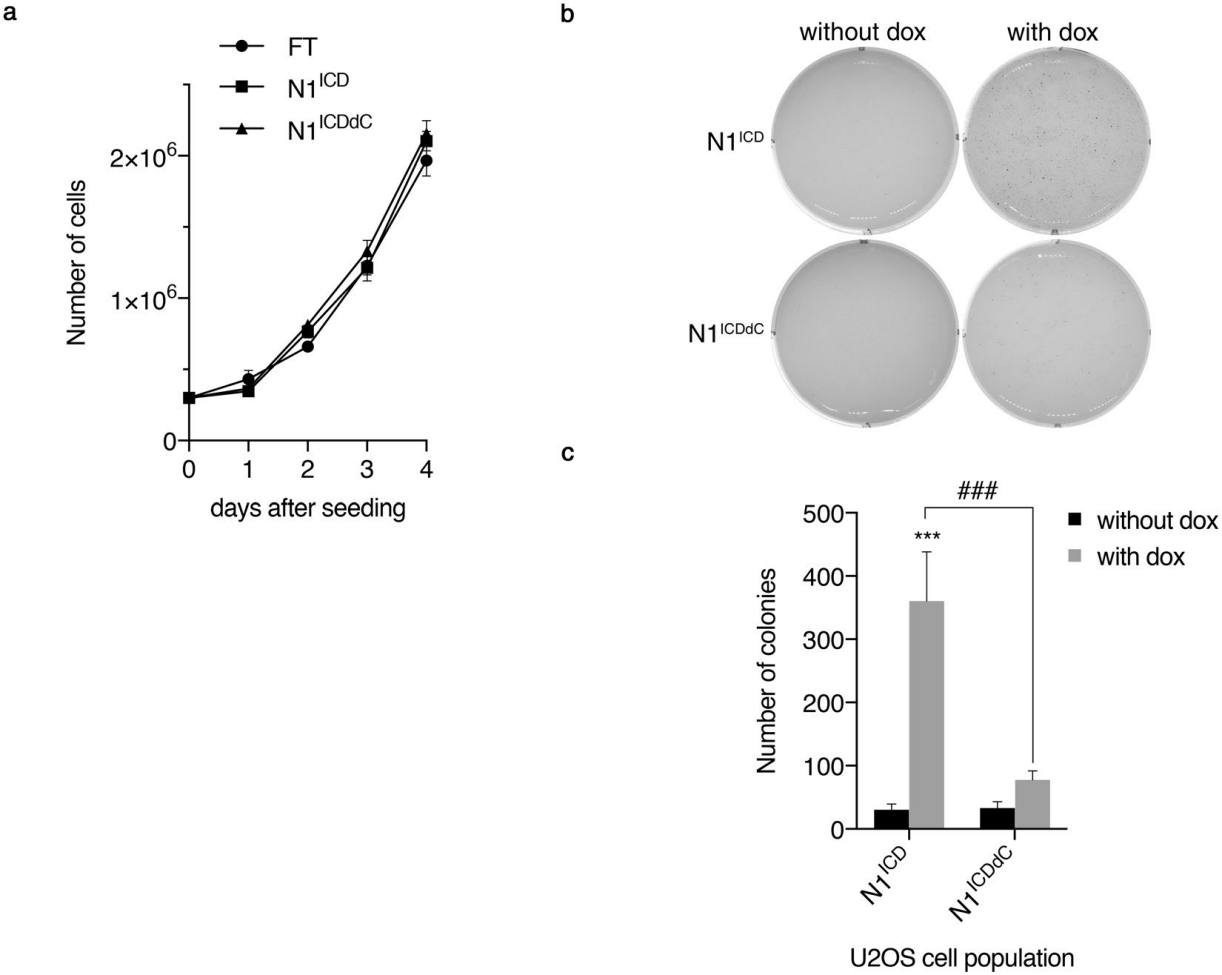
N1^ICD^ promotes anchorage-independent growth. (**a**) The parental U2OS Flp-In^TM^ T-REx^TM^ (FT) population, and stable populations of U2OS GFP-N1^ICD^ (N1^ICD^) and GFP-N1^ICDdC^ (N1^ICDdC^) were seeded on day 0 and growth was assessed by cell counting for 4 days. The experiment was performed 4 times in duplicate. Data are expressed as mean ± SEM. (**b**) Cells from stable populations of U2OS GFP-N1^ICD^ (N1^ICD^) and GFP-N1^ICDdC^ (N1^ICDdC^) were seeded in soft-agarose and left uninduced (without dox) or induced with doxycycline (with dox). After 3–4 weeks, colonies were stained. (**c**) Same as (**b**). Stained colonies were counted using the CellProfiler software. A graphical representation of 10 independent experiments is shown. ***p < 0.001 as compared with the uninduced cells (without dox). ###p < 0.001.

To determine whether the apparent distinct transcriptional potential of N1^ICDdC^ was functionally relevant, the growth properties of U2OS GFP-N1^ICD^ and U2OS GFP-N1^ICDdC^ were evaluated. The anchorage-dependent growth curve of the parental U2OS Flp-In^TM^ T-REx^TM^ cell line as well as the stable cell populations expressing either GFP-N1^ICD^ or GFP-N1^ICDdC^ were similar (Fig. 5a). To evaluate whether Notch signalling mediated by N1^ICD^ or N1^ICDdC^ could impact on a trait of transformed cells, anchorage-independent growth was assessed. Uninduced U2OS cell populations were able to form colonies in soft agarose but with limited capacity (Fig. 5b and c). Solely U2OS cells induced to express N1^ICD^ consistently and significantly formed more colonies in soft agarose as compared to their uninduced counterpart (Fig. 5b and c). So, despite lower expression levels, N1^ICD^ appears more potent than N1^ICDdC^ in promoting anchorage-independent growth of U2OS cells.

## Discussion

Many studies in the literature have exploited the Rosa^N1-ICD^ mouse strain to direct Notch1 activation in specific cell types and evaluate its impact on developmental processes or carcinogenesis. Noteworthy, upon Cre-mediated recombination, Rosa^N1-ICD^ mutant mice express a deleted version of the mouse N1^ICD^ lacking its last C-terminal 238 amino acids. Given the increasing evidence that this region harbours potential regulatory sites^8, 9^, this study was undertaken to test whether Notch1 signalling triggered by a C-terminally truncated N1^ICD^ (N1^ICDdC^) is indistinguishable from N1^ICD^-mediated signalling particularly in the context of a human N1^ICD^.

Essentially, our observations support previous data demonstrating an increased stability of N1^ICDdC^ owing to its escape from proteasomal degradation. However, we uncovered that this higher expression levels of N1^ICDdC^ is not converted into an elevated Notch-mediated transcriptional output on the *Hes1* promoter or a CSL-responsive reporter gene. The impact on *Hes1* promoter activity was mitigated since N1^ICDdC^, particularly in U2OS cells, was still able to upregulate the activity of the *Hes1*-promoter and increase HES1 protein expression levels. However, deletion of the C-terminal domain of N1^ICD^ dramatically impaired its ability to induce the activity of the Notch-dependent construct CSL-luciferase. Of note, 4X CSL binding sites are in tandem in the CSL-luciferase reporter gene whereas 2 CSL binding sites are positioned head to head on the Hes1-luciferase reporter gene^11, 25^. Altogether, our results suggest that N1^ICDdC^ might be competent in influencing a subset of Notch target genes, but is probably unable to faithfully recapitulate the repertoire of N1^ICD^ targets.

We showed that N1^ICDdC^ was still able to localize within the nucleus and associate with CSL and MAML1. This may not be surprising given that the domains involved in CSL and MAML1 interaction are still present on N1^ICDdC^ ^26–28^. Still, we cannot totally exclude the possibility that the N1^ICD^/CSL/MAML1 ternary complex could be more efficiently assembled than the N1^ICDdC^/CSL/MAML1 complex, as the stoichiometric of the proteins of the ternary complex was regularly dissimilar in the N1^ICD^ vs. N1^ICDdC^ immunocomplexes. It is also possible that solely the conformation of the N1^ICD^/CSL/MAML1 ternary complex engages additional interactors potentially modulating its transcriptional activity. In support of this possibility, we frequently observed post-translational modifications on MAML1 associated with N1^ICD^ (see Fig. 4c) suggesting that the N1^ICD^/CSL/MAML1 ternary complex, but not N1^ICDdC^/CSL/MAML1, may recruit additional regulators of the Notch core transcriptional complex. Further studies are clearly needed to identify the mechanisms orchestrating the assembly of the N1^ICD^/CSL/MAML1 complex as the regulatory events occurring after NOTCH1 cleavage/N1^ICD^ release up to its integration into a transcriptional platform remain elusive.

Our data revealed that only the forced expression of N1^ICD^ significantly promotes the anchorage-independent growth capacity of U2OS. Therefore, the extent of the transformed phenotype cannot be extrapolated from N1^ICD^ vs. N1^ICDdC^ expression levels. A previous study reported similar observation where varied levels of N1^ICD^ expression was not correlating with the extent of transformation of immortalized RKE cells^29^. One lesson from our study is thus that the Notch-induced phenotype and transcriptional output on limited but common Notch-dependent reporter genes cannot be deduced from N1^ICD^ or mutant N1^ICD^ expression levels.

Our results also imply that the Notch response is likely to be affected by the level of Notch activation. It has been shown that high expression levels of N1^ICD^ reduces cervical cancer cell proliferation by interfering with expression of the human papilloma viral oncogene E6 and E7 whereas moderate expression levels of N1^ICD^ cooperates with E6/E7 to transformed keratinocytes^30^. The extent of Notch1 activation was also showed to shape the phenotype of mammary epithelial cells^31^ as well as being determinant in influencing hematopoiesis and T-ALL initiation^32^. So, as for many signalling pathways, it is becoming clear that the amplitude and the duration of the Notch-dependent transcriptional output will impact on the cellular outcomes^1^. From our observations, it is tempting to speculate that the C-terminal domain of N1^ICD^ harbours regulatory sites that affect its function. In the same line of idea, other studies proposed a role for the C-terminal domain, not only in the stabilization of the protein, but also on the transcriptional potential of N1^ICD^. Notably, Gerhardt *et al*. demonstrated that deletion of amino acids 2193 to 2396 in mouse N1^ICD^ (amino acids 2203 to 2421 in human N1^ICD^) led to higher expression levels of N1^ICD^ but the latter had a reduced capacity to bind Notch responsive elements^33^. The generation of knock-in mice expressing this truncated form of N1^ICD^ allowed them to conclude that this region is not critical for all Notch1 functions but may play a role in enhancing or facilitating the expression of a subset of Notch1 target genes. More recently, methylation of mouse N1^ICD^ on 5 conserved arginine residues was shown to shape the Notch response^21^. Three out of these 5 arginine residues are deleted in our N1^ICDdC^ construct. It is noteworthy that methylation-defective N1^ICD^ is more stable, still associates with CSL but displays reduced transcriptional activity and is biologically less active, observations that closely remind our results. Interestingly, the authors developed a mathematical model in which they predicted that N1^ICD^ would produce a robust but short transcriptional response whereas methylation-defective N1^ICD^ would lead to a dampened but more prolonged transcriptional output. This model fits well with our findings in U2OS demonstrating that higher expression levels of N1^ICDdC^ as compared to N1^ICD^ leads to comparable expression levels of the Notch target HES1.

It is worth mentioning that Notch signalling was reported to elicit CSL-dependent and -independent cellular responses^34–36^. Notably, ChIP-seq and bioinformatics recently showed that DNA binding sites of N1^ICD^ and CSL are not entirely overlapping suggesting CSL-independent gene regulation by N1^ICD^ ^37^. Of interest, in *Drosophila*, there is indication that CSL-independent Notch-mediated signals require the C-terminal region of Notch^34, 38^. These observations thus support both CSL-dependent and -independent mechanisms involved in shaping the Notch response. Given that we mainly monitor CSL-dependent activity in our models, we cannot exclude that the cellular response to N1^ICD^ and N1^ICDdC^ expression, particularly on anchorage-independent growth, is consequent of changes in the proportion of CSL-dependent and –independent Notch signalling. Therefore, it could be interesting to perform ChIP-seq experiments along with microarrays in order to relate N1^ICD^ vs. N1^ICDdC^ vs. CSL DNA binding sites with gene regulation. These experiments could be informative in revealing differentially regulated CSL-dependent and -independent gene networks by N1^ICD^ and N1^ICDdC^.

Overall, we have generated cell models with single targeted integration of human N1^ICD^ or N1^ICDdC^ in order to test the requirement of the C-terminal domain in withstanding N1^ICD^ function. Despite leading to increased stability of N1^ICD^, deletion of the C-terminal domain did not increase the ability of N1^ICD^ to modulate some Notch responsive promoters. Furthermore, deletion of this region limited the capacity of N1^ICD^ to promote anchorage-independent growth. In light of these results, it is worth asking whether the Rosa^N1-ICD^ mouse strain^7^, expressing a truncated version of mouse N1^ICD^ similar to our human N1^ICDdC^, faithfully recapitulate the full spectrum of N1^ICD^ function. Of note, with regards to the role of Notch1 signalling during development, truncated forms of N1^ICD^ have never been detected in normal embryonic or adult tissues. C-terminally deleted forms of NOTCH1 have been identified, particularly in T-ALL^39, 40^, but not yet in solid tumours. In the future, it would be interesting to examine in greater details whether full length or truncated forms of NOTCH1, by modulating distinctly the amplitude and the duration of CSL-dependent and –independent NOTCH1 signalling, permit the optimal gene expression profile required to transform cells or promote tumour progression in a concerted action with cell context.

## Methods

### Plasmid expression constructs

The human N1^ICD^ and N1^ICDdC^ were amplified by PCR from pcDNA3-NOTCH1 expressing vector (kindly provided by Stephen C. Blacklow, Boston). The N1^ICD^ sequence encodes for amino acids 1754–2555 whereas N1^ICDdC^ encodes for amino acids 1754–2301 of human NOTCH1. The oligonucleotides included the BP recombination sites *attB*. To generate the entry clones pDONR 221-N1^ICD^ and pDONR 221-N1^ICDdC^, BP Clonase® (Life Technologies) was used for BP recombination reaction between the *attB*-containing PCR products and the *attP*-containing donor vector pDONR 221. To generate N-terminally GFP-tagged N1^ICD^ and N1^ICDdC^ expressing plasmids, LR Clonase^TM^ was used for LR recombination reaction between *attL*-containing pDONR 221-N1^ICD^ or pDONR 221-N1^ICDdC^ and *attR*-containing destination vector pDEST53 or pgLAP1^41^.

### Cell culture and treatments

The human pancreatic cancer cells MIA PaCa-2 (American Type Culture Collection) were grown in DMEM medium supplemented with 10% fetal bovine serum (FBS) and 2 mM glutamax in humidified 5% CO_2_ atmosphere at 37 °C. The U2OS Flp-In^TM^ T-REx^TM^ cell line was cultured in DMEM medium supplemented with 10% FBS, 2 mM glutamax and 100 μg/mL Zeocin and 5 μg/mL Blasticidine-HCl. Stable U2OS GFP-N1^ICD^ and GFP-N1^ICDdC^ cell populations were obtained by transfecting the U2OS Flp-In^TM^ T-REx^TM^ cell line with pgLAP1-N1^ICD^ or pgLAP1-N1^ICDdC^ plasmid along with the Flp-recombinase expressing plasmid pOG44 followed by 10-days selection with 100 μg/mL Hygromycin B and 5 μg/mL Blasticidine-HCl. Of note, for each U2OS GFP-N1^ICD^ and GFP-N1^ICDdC^, two independent stable cell populations were generated and displayed similar characteristics. Results obtained with all cell lines are presented and included within graph representation and statistical analyses.

Induction of GFP-N1^ICD^ and GFP-N1^ICDdC^ protein expression was carried out by addition of 1 μg/mL doxycycline for 24 h or the indicated time period. When indicated, cells were incubated with the protein synthesis inhibitor cycloheximide (25 μg/mL) or the proteasome inhibitor MG132 (10 μM) for the indicated time period.

### Extracts, Immunoblotting and antibodies

Cells were washed with ice-cold PBS before being lysed in Triton buffer (1% Triton X-100, 50 mM Tris pH 7.5, 100 mM NaCl, 5 mM EDTA, 0.2 mM orthovanadate, 40 mM β-glycerophosphate, 50 mM NaF, 10% glycerol, 1 mM PMSF, 0.5 μg/mL aprotinin, 0.5 μg/mL leupeptin and 0.7 μg/mL pepstatin). Total cell lysates were cleared of cellular debris by centrifugation (10 000 rpm, 10 min, 4 °C). Subcellular fractionation was performed as previously described^42^. Protein concentrations were measured using the bicinchronic acid (BCA) reagent procedure from Pierce with bovine serum albumin as standard. Equal amounts of protein were separated by sodium dodecyl sulfate-polyacrylamide gel electrophoresis (SDS-PAGE), and proteins were detected immunologically after electrotransfer onto nitrocellulose or polyvinylidene difluoride membranes as previously described^42^. The anti-GFP, anti-LAMIN B and anti-NOTCH1 antibodies were from Santa Cruz Biotechnology and anti-ACTIN from EMD Millipore. The anti-MAML1, anti-CSL, anti-cleaved NOTCH1, anti-HES1 and anti-GAPDH were from Cell Signaling Technology. Horseradish peroxidase (HRP)-conjugated anti-mouse and anti-rabbit IgG were from Jackson Immunoresearch Laboratories.

### Immunoprecipitation and phosphatase assays

Immunoprecipitation and phosphatase assays were performed essentially as previously described^18, 42^. Briefly, 1–2 mg of cleared lysates were incubated with GFP-Trap agarose beads (Chromotek) or anti-MPM2 antibody (EMD Millipore) for 2 h at 4 °C under agitation. SureBeads Protein G magnetic beads (Bio-Rad) were subsequently added to MPM2 immunocomplexes for 1 h at 4 °C under agitation. The beads were then washed thrice with lysis buffer before boiling for 5 min in 4X Laemmli sample buffer (1X = 62.5 mM Tris-HCl pH 6.8, 2.3% SDS, 10% glycerol, 1 mM PMSF, 0.005% bromophenol blue and 5% β-mercaptoethanol). For phosphatase assays, immunocomplexes were washed twice with 1X NEBbuffer pack for Protein MetalloPhosphatase (New England Biolabs Inc.) supplemented with 1 mM MnCl_2_ and 1 mM PMSF, 0.5 μg/mL leupeptin, 1 μg/mL pepstatin and 0.5 μg/mL aprotinin. The beads were then equally split in two Eppendorf tubes. 400 units of lambda protein phosphatase (New England Biolabs Inc.) were added to one tube and both tubes were incubated for 30 min at 30 °C. The reaction was stopped by adding 4X Laemmli sample buffer.

### Transient transfections and luciferase assays

Experiments were performed essentially as described previously^43^. Briefly, for transient transfection, MIA PaCa-2 cells were transfected with the indicated plasmids, lysed 24–48 h post-transfection and prepared for immunoblotting. For luciferase assays, cells were transfected with firefly-luciferase reporter construct (CSL-luciferase, gift from Nicholas Gaisano Addgene plasmid #26897^44^; Hes1-luciferase^45^, kind gift from Ruth S. Slack, Ottawa), Renilla-luciferase reporter plasmid (pRL-thymidine kinase luciferase) and an expressing vector when indicated (pDEST53, pDEST53-N1^ICD^ or pDEST53-N1^ICDdC^). Cells were harvested 24–48 h post-transfection in passive lysis buffer (Promega) and luciferase activities were determined using the Dual Luciferase Assay kit (Promega) according to the manufacturer’s instruction. The data are expressed as firefly-luciferase activity normalized to Renilla-luciferase activity.

### Cell growth and Soft Agarose Assay

For anchorage-dependent growth, 300 000 cells from U2OS Flp-In^TM^ T-REx^TM^, uninduced (without doxycycline added) U2OS GFP-N1^ICD^ or U2OS GFP-N1^ICDdC^ population were seeded in duplicate in 35 mm dishes in medium containing doxycycline. At each time point, cells were trypsinized and cell number evaluated by cell counting using a hemacytometer. Anchorage-independent growth was tested as previously described^42^. Briefly, 6-well dishes were precoated with 1.5 mL/well mixture (1:1) of DMEM 2X without phenol red and agarose type VII 1.4% (Sigma-Aldrich). Cells were then seeded on top of the precoated wells by adding 2 mL of DMEM-agarose mixture (1:1) containing 100 000 cells, after which the plates were allowed to solidify. Fresh DMEM 1X without phenol red supplemented with 10% FBS together with or without doxycycline (1 μg/mL) was added to the surface of the agarose and changed daily. After 3–4 weeks, colonies were stained by adding 500 μL of MTT (Calbiochem) at 0.5 mg/mL in PBS to the surface of the agarose and incubated for 5 h at 37 °C in 5% CO_2_. Images were acquired and colonies were counted using the CellProfiler 2.2.0 software.

### Statistical Analysis

Densitometric analyses were performed using the ImageJ software version 1.48 v. Data were analyzed by Prism 7 version 7.0a (GraphPad Software, Inc). Except for luciferase assays that were analyzed by unpaired two-tailed t-test, comparison of multiple groups was done by two-way ANOVA. Results are expressed as mean and error bars represent SEM. Differences were considered statistically significant when p < 0.05.

## Electronic supplementary material

Supplementary Information
