# Supplementary material for: C-terminal deletion of NOTCH1 intracellular domain (N1ICD) increases its stability but does not amplify and recapitulate N1ICD-dependent signalling
Source: Sci Rep. 2017 Jul 11;7:5034. doi: 10.1038/s41598-017-05119-0 (PMC5506007; doi:10.1038/s41598-017-05119-0)
Supplement: Supplementary file 1 — Supplementary Information [file 41598_2017_5119_MOESM1_ESM.pdf]

SUPPLEMENTARY INFORMATION

C-terminal deletion of NOTCH1 intracellular domain ($N1^{ICD}$) increases its stability but does not amplify and recapitulate $N1^{ICD}$-dependent signalling

Jennifer Blain, Jessily Bédard, Maureen Thompson, François-Michel Boisvert and Marie-Josée Boucher

Full-length blots for western blots displayed in Figures 1,2,3,4.

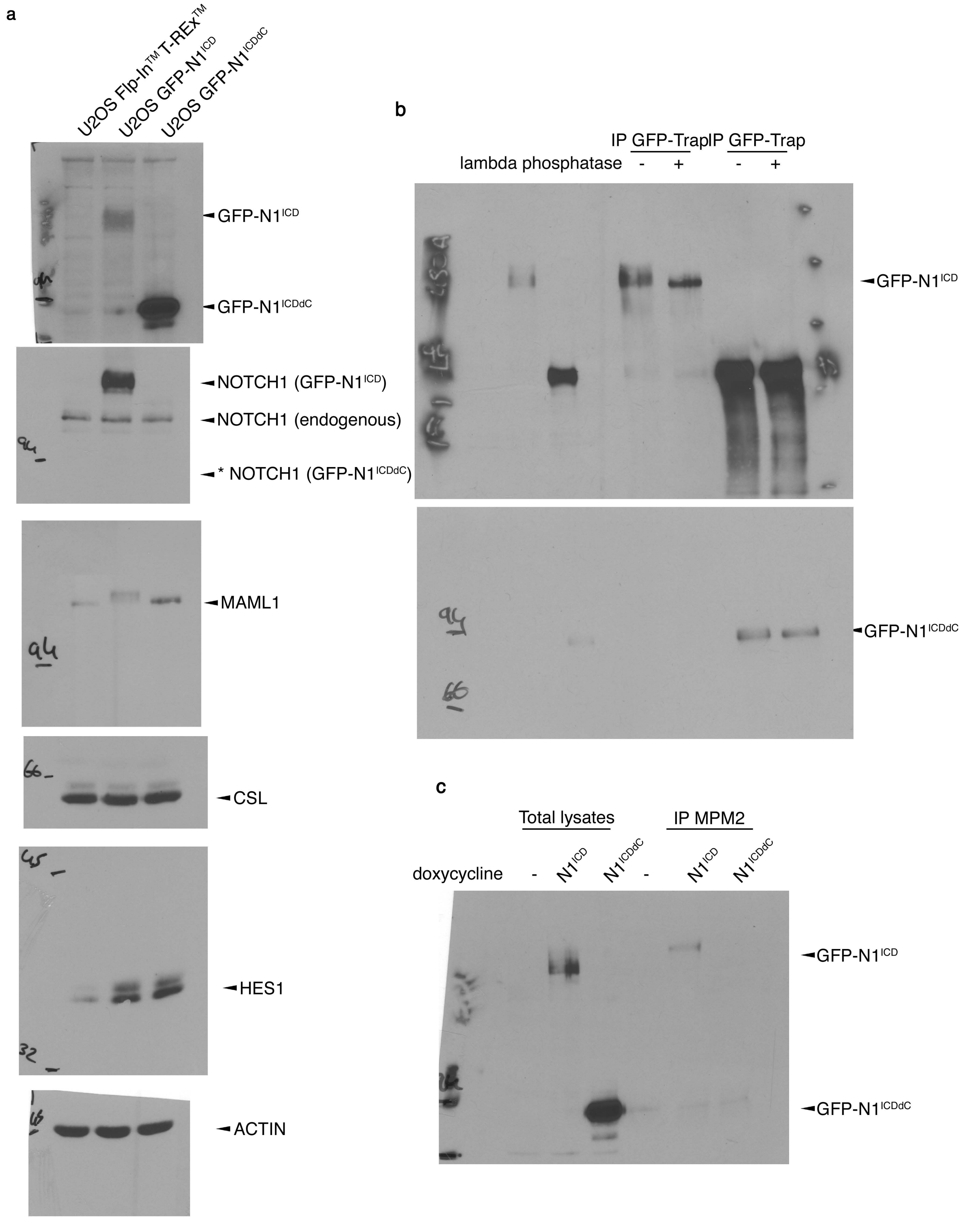
a
U2OS Flp-In^{TM} T-REx^{TM}
U2OS GFP-N1^{ICD}
U2OS GFP-N1^{ICDdC}
GFP-N1^{ICD}
GFP-N1^{ICDdC}
NOTCH1 (GFP-N1^{ICD})
NOTCH1 (endogenous)
* NOTCH1 (GFP-N1^{ICDdC})
MAML1
CSL
HES1
ACTIN
b
IP GFP-TrapIP GFP-Trap
lambda phosphatase - + - +
GFP-N1^{ICD}
GFP-N1^{ICDdC}
c
Total lysates
IP MPM2
doxycycline - N1^{ICD} N1^{ICDdC} - N1^{ICD} N1^{ICDdC}
GFP-N1^{ICD}
GFP-N1^{ICDdC}

FIGURE 1.

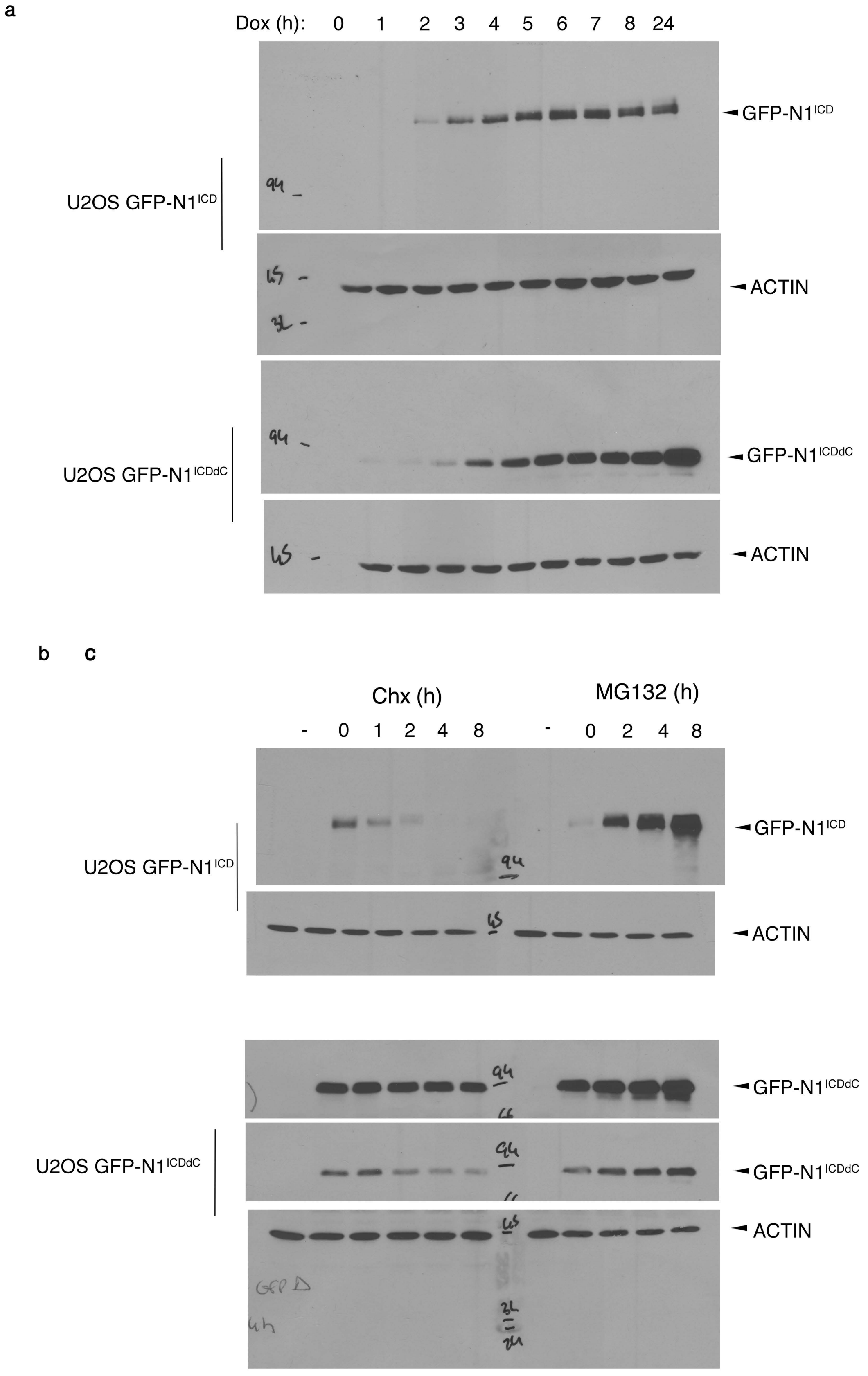
a
Dox (h): 0 1 2 3 4 5 6 7 8 24
GFP-N1ICD
U2OS GFP-N1ICD
ACTIN
U2OS GFP-N1ICDdC
GFP-N1ICDdC
ACTIN
b
c
Chx (h)
MG132 (h)
- 0 1 2 4 8
- 0 2 4 8
U2OS GFP-N1ICD
GFP-N1ICD
ACTIN
U2OS GFP-N1ICDdC
GFP-N1ICDdC
GFP-N1ICDdC
ACTIN

FIGURE 2.

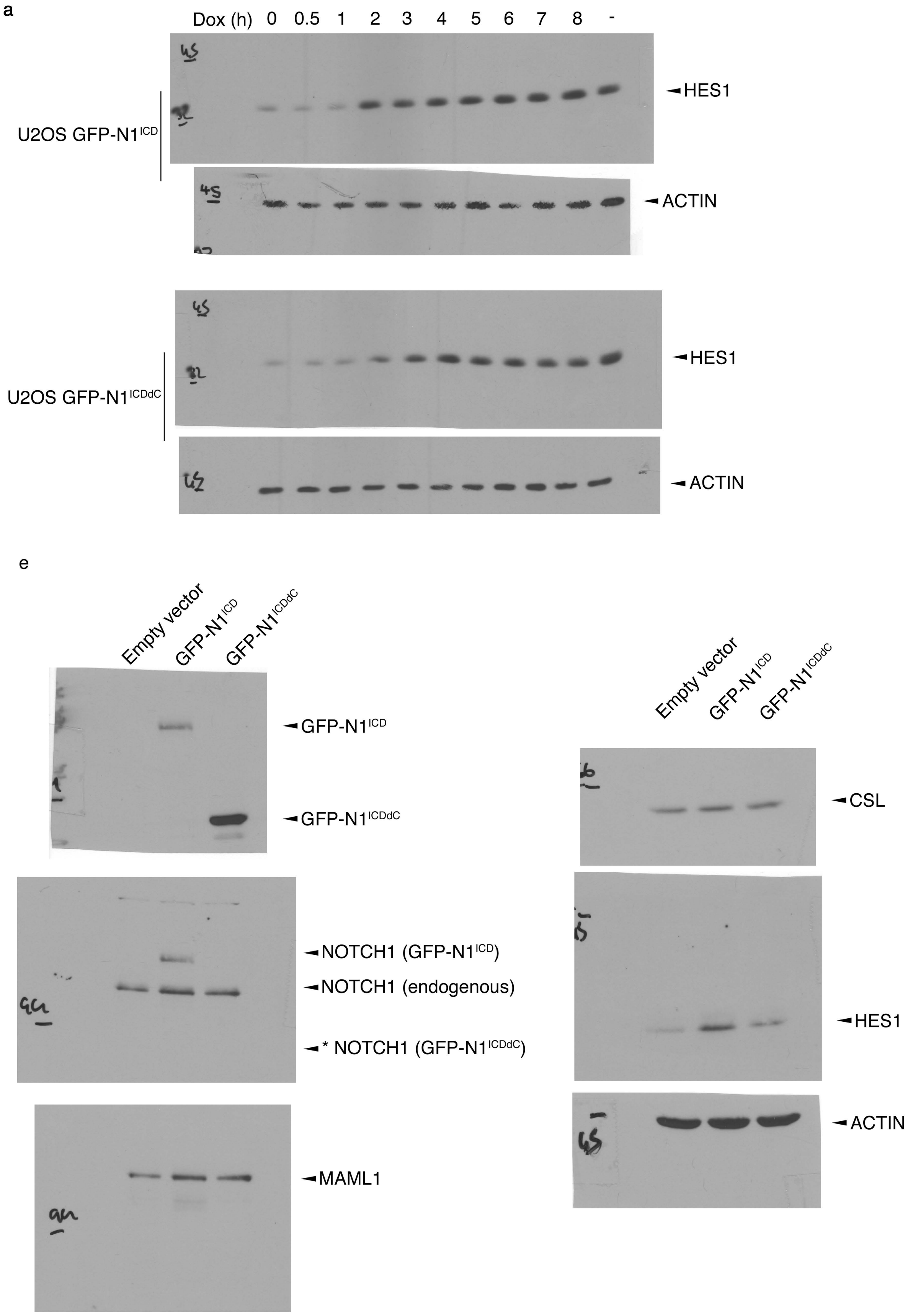
a
Dox (h) 0 0.5 1 2 3 4 5 6 7 8 -
U2OS GFP-N1 ICD
HES1
ACTIN
U2OS GFP-N1 ICDdC
HES1
ACTIN
e
Empty vector
GFP-N1 ICD
GFP-N1 ICDdC
GFP-N1 ICD
GFP-N1 ICDdC
NOTCH1 (GFP-N1 ICD)
NOTCH1 (endogenous)
* NOTCH1 (GFP-N1 ICDdC)
MAML1
Empty vector
GFP-N1 ICD
GFP-N1 ICDdC
CSL
HES1
ACTIN

FIGURE 3

a

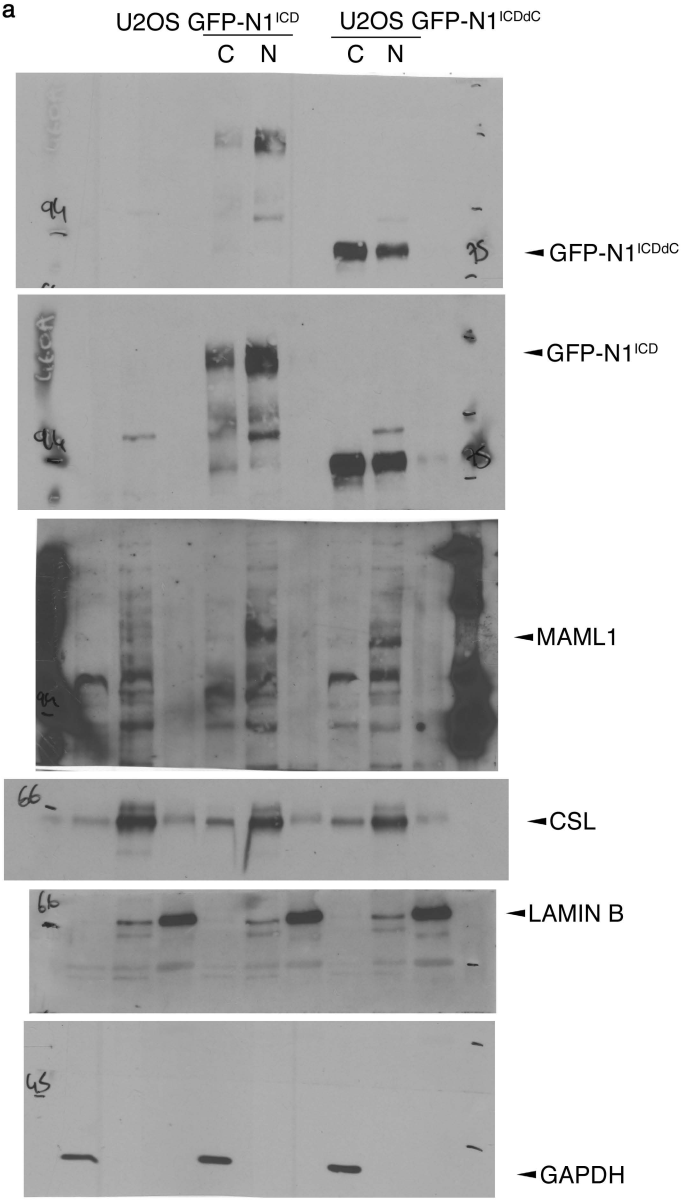

U2OS GFP-N1ICD
U2OS GFP-N1ICDdC
C
N
C
N
GFP-N1ICDdC
GFP-N1ICD
MAML1
CSL
LAMIN B
GAPDH

d

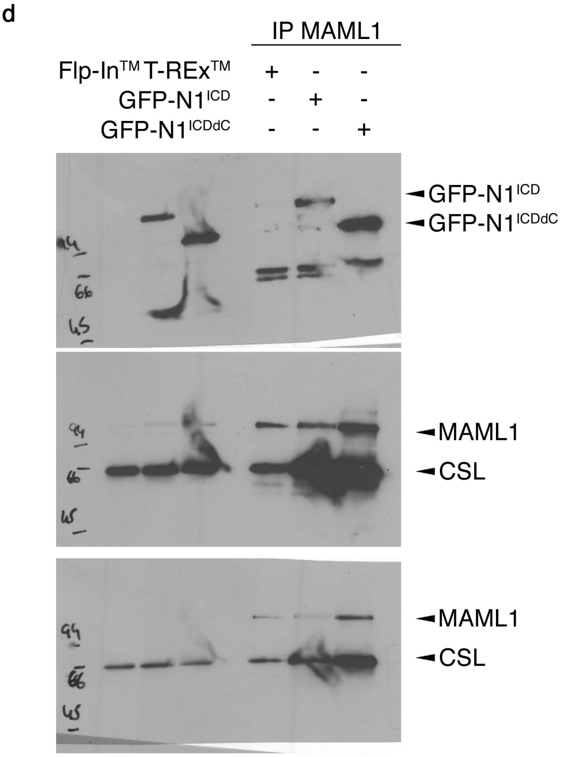

IP MAML1
Flp-InTM T-RExTM + - -
GFP-N1ICD - + -
GFP-N1ICDdC - - +
GFP-N1ICD
GFP-N1ICDdC
MAML1
CSL
MAML1
CSL

c

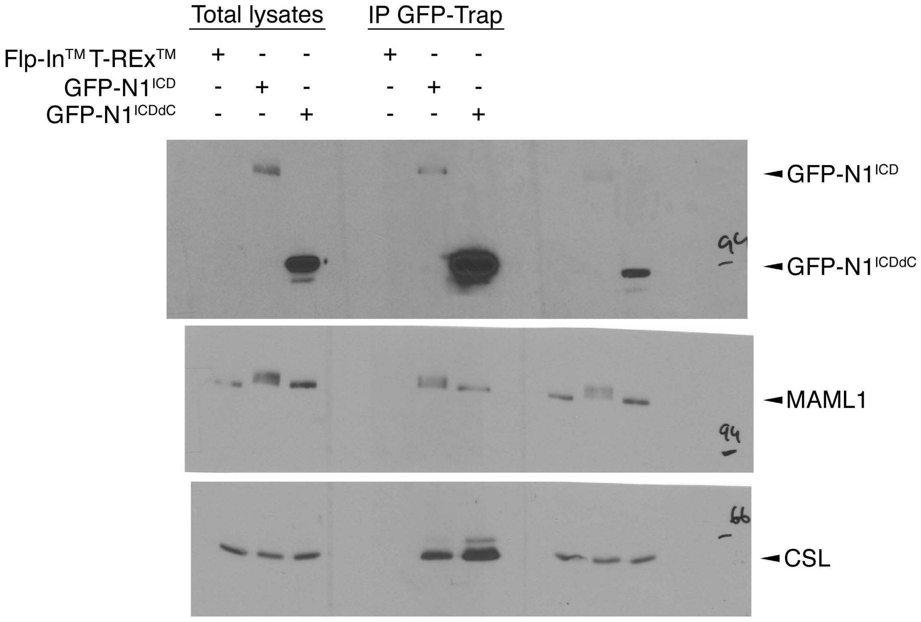

Total lysates
IP GFP-Trap
Flp-InTM T-RExTM + - - + - -
GFP-N1ICD - + - - + -
GFP-N1ICDdC - - + - - +
GFP-N1ICD
GFP-N1ICDdC
MAML1
CSL
